# Low handgrip strength is closely associated with anemia among adults: A cross-sectional study using Korea National Health and Nutrition Examination Survey (KNHANES)

**DOI:** 10.1371/journal.pone.0218058

**Published:** 2020-03-20

**Authors:** Yu-mi Gi, Boyoung Jung, Koh-Woon Kim, Jae-Heung Cho, In-Hyuk Ha

**Affiliations:** 1 Jaseng Hospital of Korean Medicine, Gangnam-gu, Seoul, Republic of Korea; 2 Department of Health Administration, Hanyang Women’s University, Seongdong-gu, Seoul, South Korea; 3 Department of Korean Rehabilitation Medicine, College of Korean Medicine, Kyung Hee University, Dongdaemun-gu, Seoul, Republic of Korea; 4 Department of Korean Rehabilitation Medicine, College of Korean Medicine, Kyung Hee University, Dongdaemun-gu, Seoul, Republic of Korea; 5 Jaseng Spine and Joint Research Institute, Jaseng Medical Foundation, Gangnam-gu, Seoul, Republic of Korea; Addis Ababa University School of Public Health, ETHIOPIA

## Abstract

**Background:**

Anemia, which is a major public health problem worldwide, represents a decline in the oxygenation function, and can therefore be related to low strength. However, hemoglobin cannot repair muscles directly, but is beneficial only in a supportive role. Previous studies on the relationship between handgrip strength and anemia have been controversial. Thus, we aimed to analyze the association between handgrip strength and anemia in Korean adults.

**Methods:**

This cross-sectional study used the 2013–2017 data from the 6th and 7^th^ Korean National Health and Nutrition Examination Survey (KNHANES) that included 16,638 Korean adults, aged ≥19 years, who met the inclusion or exclusion criteria. Differences in sociodemographic factors (sex, age, education, income, and employment), lifestyle factors (alcohol consumption, smoking, and physical activity), and illness and health factors [body mass index (BMI), vitamin intake, iron intake, comorbid illnesses, and handgrip strength] by existence of anemia, were analyzed using the Chi square test. Binary logistic regression analysis was used to measure the association between handgrip strength and anemia, while adjusting for other possible confounders. Subgroup analysis, stratified by sex and age, was performed.

**Results:**

Among Korean adults aged ≥19 years, 745,296 (7.7%) had anemia. A higher odds ratio, adjusted for other covariates/factors (OR) of anemia occurred in the weak handgrip strength group than in the strong handgrip strength group (OR = 1.92, 95% CI: 1.58–2.33). The subgroup analysis showed a higher OR adjusted for other covariates/factors of anemia in the weak handgrip strength group than in the strong handgrip strength group, regardless of sex or age. However, the association was greater for males (OR = 2.13, 95% CI: 1.35–3.34) and for those aged ≥65 years (OR = 1.92, 95% CI: 1.42–2.58).

**Conclusion:**

This study showed a strong association between handgrip strength and anemia that was particularly strong for males and those aged ≥65 years.

## Introduction

Anemia occurs because of insufficient hemoglobin (Hb), which provides oxygen to the body. It is one of the major clinical problems affecting approximately 12% of the elderly, globally [1]. The World Health Organization (WHO) defines anemia as Hb values below 12 g/dl for females and 13 g/dl for males [2]. Anemia has an influence on mortality rate [3], disability [4], decline in physical activity level [4, 5], and decline in the quality of life [6], in the elderly. Furthermore, since anemia has a close relationship with many illnesses, it is recommended that when the Hb level is lower than the normal, it should always be followed-up clinically [5]. Hence, the prevalence of anemia can then be reduced by appropriate treatment or by preventing the underlying illnesses that are related to the aging process [7].

Muscle weakness is related to poor physical activity and incident mobility limitations in the elderly [3, 6, 8–11]. Handgrip strength, a simple, cost-effective tool used to measure strength, is utilized in the diagnosis of muscle weakness in epidemiological studies [10]. Handgrip strength can be used as a valuable tool for the screening of patients with anemia in the context of health services. [12]. Some research has reported that handgrip strength in middle-age (45–65 years) is related to functional limitations and disabilities at least 25 years later [3, 11, 13]. Therefore, handgrip strength has been considered a factor that predicts healthy aging [11]. Handgrip strength is also related to the strength of other muscle groups, and therefore can be a good index to represent overall strength [14]. Therefore, low handgrip strength signifies low muscle mass and poor clinical mobility [15].

Decline in muscle strength and muscle density is also associated with anemia [16], which results in the decline of mobility, self-care, and usual activities. Aging is associated with strength deficits that are related to frailty, loss of independence, and physical disability [17], and anemia is commonly related to aging. Oxidative stress is one of the most important mechanisms to explain the occurrence of age-related illnesses [18] including the decline in strength [19]. Because Hb concentration plays an important role in the oxidation of blood [20, 21], anemia, defined by Hb, represents a decline in the oxygenation function and can therefore be related to low strength [4].

Previous studies on the relationship between handgrip strength and anemia have been controversial. Alley et al. [22] found a direct relationship between reduction in Hb level and the reduction in handgrip strength in the elderly Australian [4]. Anemic patients had significantly lower handgrip strength than patients without anemia aged ≥65 years. In a study by Santos et al., which included 109 elderly participants, it was also concluded that grip could be used as a tool for screening for anemia in the elderly female population [12]. On the other hand, as Joosten et al. [23] reported that a study of the 109 elderly populatithat ons, also concluded that grip could be used as a tool for screening anemia in the elderly female population and anemia did not affect grip or gait speed in inpatient studies. In previous studies, the mechanism underlying this was unclear. Moreover, research on the association between handgrip strength and anemia in Korean adults has provided insufficient data for a clear conclusion. Therefore, in this study, we aimed to investigate the association between handgrip strength and anemia based on the data from the 6^th^ and 7^th^ Korean National Health and Nutrition Examination Survey (KNHANES).

## Materials and methods

### Study design and population

The present study used the data from the 6^th^ and 7^th^ KNHANES that was conducted from January 2013 to December 2017 [19]. KNHANES is a 3-yearly cross-sectional research, which includes examinations, health surveys, and nutritional surveys, and involves a representative sample of the entire Korean population, using a stratified cluster-sampling method.

The KNHANES 6^th^ and 7^th^ were conducted by the Korea Center for Disease Control and Prevention (KCDC). All survey protocols were approved by the institutional review board (IRB) of the KCDC (approval numbers: 2013-07CON-03-4C, 2013-12EXP-03-5C, and 2015-01-02-6C). Informed consent was obtained from all participants when the surveys were conducted. Original data are publicly and freely available at the KNHANES website (http://knhanes.cdc.go.kr) for purposes such as academic research. IRB approval was not required because the study did not deal with any sensitive information, but rather accessed only publicly available data from the KNHANES (JASENG IRB File No. 2019-03-009).

**Fig 1:**
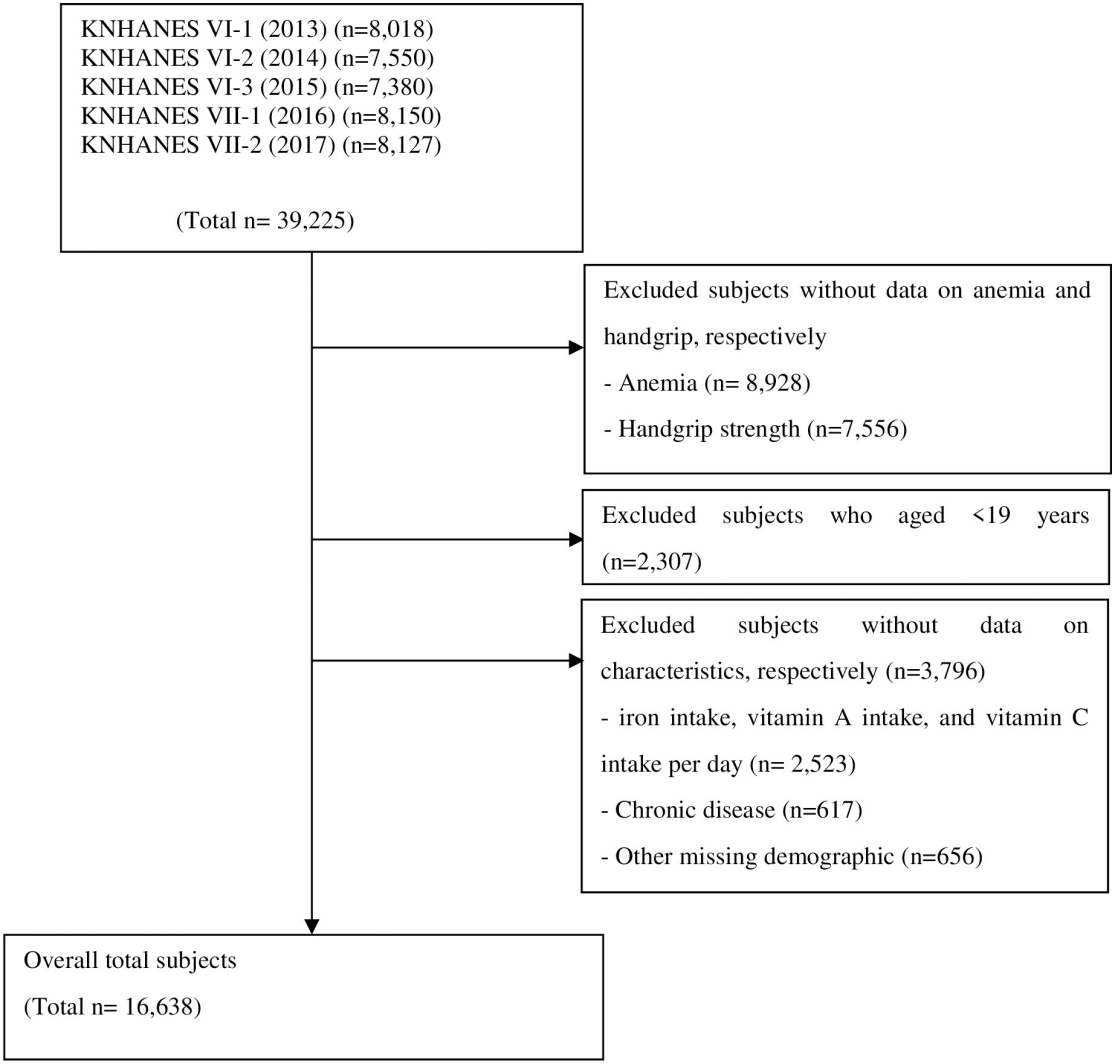
Participants flow diagram.

The KNHANES study sample consisted of Korean citizens (residents in group facilities, such as nursing homes, military, and prison as well as foreigners were excluded). To increase the representativeness of the sample and the accuracy of assumptions, sampling was designed to distinguish between province and city, neighborhood, towns, and townships, and the type of home, to extract data from sampled regions (investigated district). We included the 2013–2017 data in the handgrip strength data analysis. The exclusion criteria were as follows: those with missing data on anemia (n = 8,928) and on handgrip strength (n = 7,556), those aged under 19 years (n = 2,307), and other missing data (n = 3,797). Ultimately, the analyses were performed using data of 16,638 participants.(Fig 1).

## Outcomes and other variables

Korea National Health and Nutrition Examination Survey (KNHANES) is conducted to evaluate the health and nutritional status of the Korean people. Twenty households were selected throughout 192 regions each year and 10,000 individuals aged 1 year and over were targeted for the KNHANES. Survey components were divided in 3 parts: Health Examination, Health Interview, and Nutrition Survey[24] (https://knhanes.cdc.go.kr/knhanes/eng/index.do).

### Measurement of handgrip strength

Handgrip strength, to assess muscle strength, was obtained using a handgrip strength test in a health examination, according to the recommendations of the Institute of Medicine [14]. For the standard value of handgrip strength, we used the standard for the skeletal muscle mass (26 kg for men, 16 kg for women) suggested by the North American Foundation for the National Institutes of Health Sarcopenia Project published in 2015 [22]. The handgrip strength for each hand was measured three times using a digital grip strength dynamometer (TKK 5401; Takei Scientific Instruments Co, Ltd, Tokyo, Japan) [25]. Trained medical technicians instructed the study participants while in a sitting position to hold the dynamometer with the distal interphalangeal finger joints at a 90° angle to the handle, and to squeeze the handle as firmly as they could. Another handgrip strength measurement was done during expiration, after the participant had slowly stood up. Study participants conducted 3 attempts per hand, with a 1-minute rest period between each attempt to reduce the effect of fatigue due to repetition. The value for the handgrip strength measurements was the average of the three measurements, for either hand [26]. Using this mean handgrip strength values, participants were categorized into the weak (<26 kg for men, <16 kg for women) and strong (≥26 kg for men, ≥16 kg for women) groups.

### Measurement of Hb levels

Blood analysis that included Hb level measurements was performed and used as an index to determine the level of anemia. The hemoglobin level was measured via the cyanide-free sodium lauryl sulphate method using XE-2100D (Sysmex, Kobe, Japan) [27]. In accordance with the WHO standard, male and female participants with Hb less than 13 g/dL and 12 g/dL, respectively, were defined as anemic patients [13].

### Description of demographic and other characteristics of the study population

We analyzed the general characteristics, socioeconomic background characteristics, and lifestyle habits of the patients. The BMI, a value derived by dividing the weight with the square of height, was categorized into <18.5 kg/m^2^ (underweight), 18.5–24.9 kg/m^2^ (normal), and ≥25.0 kg/m^2^ (overweight) [18]. For smoking, participants were categorized as nonsmokers, ex-smokers, or current smokers. The frequency of alcohol consumption was divided into four categories (‘none,’ ‘less than 1 time per month’, ‘1–4 times per month’, and ‘more than 5 times per month.’) For employment, participants were categorized into ‘unemployed (student, housewife, etc.)’ or ‘employed.’ For marital status, participants were categorized into three groups (‘married-cohabiting’ for those who had spouses or cohabiting partners; ‘married-non cohabiting, bereaved, or divorced’ for those who were living apart, bereaved, divorced; or ‘unmarried’ for those who were unmarried). Household income was categorized into four groups (low, low-moderate, moderate-high, and high). The criteria used in dividing them into four groups were based on the income quartile threshold for the sample household and sample population. This is the income level divided into four groups by gender and age according to the average monthly household equalization income [28, 29].

Educational level was also categorized into four groups (elementary school or less, middle school, high school, and college or higher). Level of daily workout referred to the frequency of strength exercise per week, and was categorized into four groups (‘none’; ‘light’ [1 day]; ‘moderate’ [2~3 days]; and ‘heavy/extreme’ [4 days or more]).

### Measurement and classification of other variables of interest

The nutritional information used in our research was obtained from the food intake nutrition surveys collected by dieticians who visited the homes of participants. The survey method was a face-to-face interview within the home. The survey inquired about topics such as dietary behavior, dietary supplement use, food security, food frequency, food and dietary intake. The food frequency questionnaire is composed of 63 food items that represent key sources of energy and nutrients. The questionnaire is designed as an open-ended survey for reporting various dishes and foods; the 24-h recall method was used with various measuring aids. We obtained data on vitamin A and C intake based on the reported information collected using the food intake questionnaire [24]. For nutritional characteristics, iron intake, vitamin A intake, and vitamin C intake per day were categorized as ‘insufficient’ if the intake was lower than the recommended level by sex and age and ‘sufficient’ if the intake was above the recommended level. All blood samples were drawn after fasting and were analyzed within 24 hours of being drawn. The 2015 standards of the Dietary Reference Intakes for Koreans (KDRIs) were used for iron and vitamin C intake per day. For vitamin A intake, the 2010 KDRIs standards [30] were used with identical units (μg RE/day) for KHANES and KDRIs [31] (μg RAE/day), in 2015. Vitamin A intake per day for males and females aged 19–49 and ≥50 years were 750 and 700 μg RE/day; and 650 and 600 μg RE/day, respectively. Vitamin C intake per day was 100 mg for both males and females over 19 years old. Iron intake per day for males was 10 and 9 mg/day for those aged 19–64 and ≥65 years, respectively; while for females aged 19–49, 50–74, and ≥75 years, was 14, 8, and 7 mg/day, respectively.

Hypertension, cancer, hyperlipidemia, cardiovascular disease, kidney disease, bone disease, and thyroid disease were the comorbid diseases included. Participants were categorized into a ‘yes’ or ‘no’ group based on the diagnosis of at least one of these diseases.

## Statistical analysis

Because KNHANES data were obtained using a complex sampling design, all statistical analysis methods used complex sample analyses considering weight, stratified variables, and cluster sampling. We selected factors associated with anemia for the statistical adjustment, from previous studies. Differences in sociodemographic factors (gender [32, 33], age [34], region [35], marital status [36], income [37], employment [38], and education [39]), lifestyle factors (alcohol consumption and smoking [40], and physical activity [4, 41]), and illness and health-related factors (BMI [42], vitamin intake [43], iron intake [44–47], comorbid illnesses [48–52], and handgrip strength [12]); due to the presence of anemia, were analyzed using a complex sample analysis by Chi square (*χ*^2^) test. Categorical variables were presented as proportions (n, %) using the *χ*^2^ test of independence, while continuous variables were presented with the estimated value ± standard error (SE).

A Chi-square test was performed on risk factors associated with anemia. Furthermore, to identify factors associated with anemia, the odds ratio (OR) and 95% confidence intervals (95% CI) were obtained through a binary logistic regression analysis after setting anemia as the dependent variable and handgrip strength as the main independent variable, while controlling for sociodemographic factors, lifestyle factors, and illness and health-related factors.

The equations of the logistic regressions are as below:

$$F_{0i}=\text{log}\frac{p_{i}}{1-p_{i}}=\beta_{0i}+\beta_{1i}{Hand\;grip}_{i}\;\ldots \;(crude)$$

$$\begin{array}{c} F_{1i}=F_{0i}+\beta_{2i}{Gender}_{i}+\beta_{3i}{Age}_{i}+\beta_{4i}{Marital\;status}_{i}+\beta_{5i}{Region}_{i}+\beta_{6i}{Income}_{i}\\ +\beta_{7i}{Employment}_{i}+\beta_{8i}{Education\;level}_{i}\ldots \;(model\;1) \end{array}$$

$$\begin{array}{c} F_{2i}=F_{1i}+\beta_{9i}{Alcohol\;consumption}_{i}+\beta_{10i}{Smoking}_{i}+\beta_{11i}{Stress\;level}_{i}\\ +\beta_{12i}{Level\;of\;daily\;workoutg}_{i}\;+\beta_{13i}{BMI}_{i}\;\ldots \;(model\;2) \end{array}$$

$$\begin{array}{c} F_{3i}=F_{2i}+\beta_{14i}{Other\;diseases\;}_{i}+\beta_{15i}{Iron\;intake}_{i}+\beta_{16i}{\;Vitamin\;A\;intake\;}_{i}\\ +\beta_{17i}{\;Vitamin\;C\;intake\;}_{i}\;\ldots \;(model\;3) \end{array}$$

where *p*_*i*_ is the probability that each individual i develops dementia. The association between handgrip strength and anemia was subgroup-analyzed by gender (male/female) and age (younger or older than 65 years). All statistical analyses were performed using SPSS version 25.0 (SPSS Inc., Chicago, IL, USA) and SAS version 9.4 (SAS Institute Inc, Cary, NC), and the significance level of all tests was defined as a p-value less than 0.05.

## Results

Table 1 shows the general characteristics of the participants. Overall, 7.7% of patients had anemia. About half (51.2%) of the participants were female, of these, 11.9% had anemia. Overall, 6.7% and 15.8% of participants were in the strong and weak handgrip strength groups, respectively (p<0.001). The mean age of participants with anemia was higher (50.27 years) than that of those in the group without anemia (46.14 years) (p<0.001). According to the individual lifestyle habits and the level of daily workout, those in the light (1 day) daily workout group (615,680 participants, 8.5%) had anemia, and had a higher prevalence of anemia than those in the moderate (5.7%) and heavy/extreme (4.9%) daily workout groups (p<0.001). For the nutritional and disease-related factors, the prevalence rate of anemia in the group with comorbid diseases was 2.5% higher than in the group without comorbid diseases (p<0.001). For iron intake, the prevalence rate of anemia was 3.5% greater in the insufficient group than in the sufficient group (p<0.001).

**Table 1:** Description of the basic characteristics of the study participants.

Class		N	(%)
Total		16638	(100.0)
Handgrip (kg)-2Q	Weak	2075	(10.1)
	Strong	14563	(89.9)
Gender	Male	7133	(48.8)
	Female	9505	(51.2)
Age (years)	<65	12467	(74.9)
	≥65	4171	(25.1)
Region	Urban area	13551	(84.6)
	Rural area	3087	(15.4)
Marital status	Married-cohabiting	11797	(66.7)
	Married-no cohabiting, bereaved or divorced	2177	(10.1)
	Unmarried	2664	(23.2)
Income	Low	3973	(24.6)
	Lower middle	4157	(24.7)
	Higher middle	4230	(25.1)
	High	4278	(25.6)
Employment	Unemployed	6706	(36.3)
	Employed	9932	(63.7)
Education level	Elementary school or less	3567	(15.0)
	Middle school	1722	(8.6)
	High school	5404	(35.8)
	College or higher	5945	(40.6)
Alcohol consumption	Never drink	4569	(23.0)
	Less than 1 time per month	3074	(18.1)
	1–4 times per month	5395	(35.7)
	≥5 drinking episodes per month	3600	(23.2)
Smoking	Non-smoker	10202	(58.1)
	Past smoker	3535	(21.2)
	Current smoker	2901	(20.7)
Level of daily workout	Light	12747	(74.4)
	Moderate	1348	(9.2)
	Heavy/extreme	2543	(16.4)
Stress level	High	4229	(27.4)
	Middle	9447	(57.3)
	Low	2868	(14.9)
	Zero	94	(.4)
BMI (kg/m2)	Normal weight	10385	(62.1)
	Underweight	648	(4.3)
	Obese	5605	(33.7)
Other diseases †	No	9665	(65.8)
	Yes	6973	(34.2)
Iron intake per day (mg/day)	Insufficiency	5901	(37.2)
	Sufficiency	10737	(62.8)
Vitamin A intake per day (μg RE/day)	Insufficiency	11924	(72.6)
	Sufficiency	4714	(27.4)
Vitamin C intake per day (mg/day)	Insufficiency	11492	(71.0)
	Sufficiency	5146	(29.0)

* Number is the unweighted N while percent is the weighted %

^†^ Other diseases include hypertension, cancer, hyperlipidemia, cardiovascular disease, kidney disease, bone disease, and thyroid disease

Abbreviations: SD, standard deviation; BMI, body mass index;

Table 2 shows the description of the anemia status of the study participants and stratification based on gender and age. Anemia prevalence rate was higher in females (11.9%) and in participants ≥65 years (12.7%) (p<0.001, Table 2).

**Table 2:** Description of anemia status among the study participants.

Class		Number (n)	Percent (%)	Anemia *				P-value†
				No		Yes		
				Number (n)	Percent (%)	Number (n)	Percent (%)	
Gender	Male	7133	48.8	6,776	96.8	357	3.2	p<0.001
	Female	9505	51.2	8,418	88.1	1,087	11.9	
Age (65 years)	<65	12467	74.9	11,551	92.7	916	7.3	p<0.001
	≥65	4171	25.1	3,643	87.3	528	12.7	

^†^Chi-Square test was performed to determine differences between groups with/without anemia.

* Number is the unweighted N while percent is the weighted %

Table 3 shows the description of handgrip strength in the entire participants, and stratification based on age, sex, and anemia status. Handgrip prevalence rate was higher in females (87.8%), in participants <65 years (94.4%) and in participants without anemia (88.7%) (p<0.001, Table 3).

**Table 3:** Description of handgrip strength in the entire participant group.

		Patient number (case)	Handgrip (kg)-2Q *				P-value†
			weak		strong		
			Number (n)	Percent (%)	Number (n)	Percent (%)	
Total		**16638**	2075	12.5	14563	87.5	
Gender	Male	7133	918	12.9	6215	87.1	<0.0001
	Female	9505	1157	12.2	8348	87.8	
Age (65 years)	1–64	12467	701	5.6	11766	94.4	<0.0001
	>65	4171	1374	32.9	2797	67.1	
Anemia	No	15194	1721	11.3	13473	88.7	<0.0001
	Yes	1444	354	24.5	1090	75.5	

^†^ Chi-Square test or t-test was performed to determine differences between weak and strong.

* Number is the unweighted N while percent is the weighted %.

Table 4 shows the association between handgrip strength and anemia using logistic regression analyses. The OR (odds ratio) for weak handgrip strength compared to strong handgrip strength was 2.02, 1.98, and 1.92 in Models 1, 2, and 3, respectively, showing a significant association between weak handgrip strength and anemia (p<0.001). The OR of underweight and overweight BMI was higher than of the normal weight, and the OR was also higher in persons with other diseases. Moreover, the prevalence of anemia was higher in persons with insufficient iron, vitamin A, and vitamin C intake per day than in persons with sufficient intake (Table 4).

**Table 4:** Association between handgrip and anemia.

Category		Model 1				Model 2				Model 3			
		OR	95% CI		P-value	OR	95% CI		P-value	OR	95% CI		P-value
**Handgrip**	**weak**	**2.02**	**1.67**	**2.45**	**<0.001**	**1.98**	**1.63**	**2.40**	**<0.001**	**1.92**	**1.58**	**2.33**	**<0.001**
	**strong**	**1.00**				**1.00**				**1.00**			
Gender	Male												
	Female	2.45	1.94	3.10	<0.001	2.22	1.71	2.88	<0.001	2.08	1.60	2.70	<0.001
Age(years)		1.01	1.00	1.02	0.003	1.01	1.00	1.02	0.029	1.01	1.00	1.02	0.021
Region	Urban area	1.00	0.82	1.23	0.978	1.01	0.82	1.24	0.954	1.01	0.82	1.24	0.939
	Rural area												
Marital status	Married-cohabiting	1.33	1.01	1.74	0.041	1.37	1.04	1.80	0.027	1.40	1.06	1.84	0.019
	Married-no cohabiting or bereaved or divorced	1.23	0.88	1.70	0.221	1.33	0.95	1.85	0.093	1.33	0.95	1.86	0.091
	Unmarried	1.00				1.00				1.00			
Income	Low	1.27	1.04	1.56	0.020	1.29	1.05	1.59	0.014	1.26	1.02	1.55	0.030
	Lower middle	1.52	1.27	1.83	<0.001	1.52	1.27	1.83	<0.001	1.50	1.24	1.80	0.000
	Higher middle	1.18	0.97	1.43	0.090	1.19	0.98	1.44	0.083	1.16	0.96	1.42	0.125
	High	1.00				1.00				1.00			
Employment	Unemployed	1.06	0.91	1.22	0.461	0.97	0.84	1.12	0.668	0.98	0.85	1.13	0.764
	Employed	1.00											
Education level	Elementary school or less	0.69	0.55	0.86	0.001	0.74	0.59	0.94	0.013	0.71	0.56	0.91	0.006
	Middle school	0.60	0.46	0.79	<0.001	0.64	0.49	0.84	0.001	0.64	0.49	0.85	0.002
	High school	0.98	0.83	1.15	0.796	1.02	0.87	1.20	0.791	1.02	0.87	1.20	0.831
	College or over	1.00				1.00				1.00			
Alcohol consumption	Never drink					1.49	1.21	1.84	<0.001	1.52	1.23	1.87	<0.001
	Less than 1 time per month					1.39	1.10	1.76	0.006	1.42	1.12	1.80	0.003
	1–4 times per month					1.26	1.03	1.56	0.026	1.28	1.04	1.58	0.019
	≥5 drinking episodes per month					1.00				1.00			
Smoking	Non-smoker					1.60	1.20	2.13	0.001	1.62	1.22	2.16	0.001
	Past smoker					1.95	1.46	2.60	0.000	1.94	1.45	2.59	<0.001
	Current smoker					1.00				1.00			
Stress level	High					0.78	0.33	1.82	0.557	0.76	0.32	1.81	0.537
	Middle					0.80	0.34	1.87	0.601	0.79	0.33	1.87	0.586
	Low					0.82	0.35	1.93	0.645	0.81	0.34	1.94	0.634
	Zero					1.00				1.00			
Level of daily workout	Light					1.26	1.01	1.57	0.044	1.25	1.00	1.56	0.054
	Moderate					1.14	0.82	1.58	0.433	1.15	0.83	1.60	0.392
	Heavy/extreme					1.00				1.00			
BMI (kg/m2)	Normal weight					1.00				1.00			
	Underweight					1.40	1.20	1.63	<0.001	1.41	1.21	1.65	<0.001
	Obese					1.74	1.25	2.44	0.001	1.77	1.26	2.48	0.001
Other diseases †	No												
	Yes									1.12	0.95	1.31	0.176
Iron intake per day(mg/day)	Insufficiency									1.42	1.22	1.65	<0.001
	Sufficiency									1.00			
Vitamin A intake per day(μg RE/day)	Insufficiency									1.02	0.87	1.21	0.785
	Sufficiency									1.00			
Vitamin C intake per day(mg/day)	Insufficiency									1.03	0.90	1.19	0.632
	Sufficiency									1.00			

Logistic regression analysis with complex sampling design was performed by adjusting for covariates. Odds ratios indicates odds ratio; 95% CI, 95% confidence interval.

Model 1 was adjusted by sex, age, region, marital status, income, employment, education

Model 2 was adjusted by Model 1 + alcohol consumption, smoking, level of daily workout, stress level and BMI

Model 3 was adjusted by Model 2 + other disease, iron intake per day, vitamin A intake per day, and vitamin C intake per day

^†^ Other diseases include hypertension, cancer, hyperlipidemia, cardiovascular disease, kidney disease, bone disease, and thyroid disease.

Table 5 demonstrates the association between handgrip strength and anemia stratified by sex and age. Regardless of sex and age, there was a significant association between handgrip strength and anemia. In particular, the trend of weak handgrip strength in those with anemia compared to those without anemia was more pronounced in males than in females (OR: 2.13 p = 0.001, 95% CI: 1.35–3.34); and in those aged ≥65 years compared to those <65 (OR: 1.92 p<0.001, 95% CI: 1.42–2.58) (Table 5).

**Table 5:** Association between handgrip and anemia by sub-group.

Class	Category		Model 1				Model 2				Model 3			
			OR	95% CI		P-value	OR	95% CI		P-value	OR	95% CI		P-value
Male	Handgrip	Weak	2.36	1.53	3.63	<0.001	2.11	1.34	3.31	0.001	2.13	1.35	3.34	0.001
		Strong	1.00				1.00				1.00			
Female	Handgrip	Weak	1.79	1.41	2.27	<0.001	1.77	1.40	2.25	<0.001	1.71	1.35	2.16	<0.001
		Strong	1.00				1.00				1.00			
Age <65	Handgrip	Weak	1.59	1.18	2.15	0.003	1.58	1.17	2.14	0.003	1.57	1.16	2.12	0.003
		Strong	1.00				1.00				1.00			
65≤ age	Handgrip	Weak	1.97	1.47	2.64	<0.001	1.90	1.41	2.55	<0.001	1.92	1.42	2.58	<0.001
		Strong	1.00				1.00				1.00			

Logistic regression analysis with a complex sampling design was performed by adjusting for covariates. OR indicate odds ratio; 95% CI, 95% confidence interval.

Model 1 was adjusted for sex, age, region, marital status, income, employment, education

Model 2 was adjusted for Model 1 + alcohol consumption, smoking, level of daily workout, stress level and BMI

Model 3 was adjusted for Model 2 + other disease, iron intake per day, vitamin A intake per day, and vitamin C intake per day

## Discussion

This study analyzed the association between handgrip strength and anemia using representative, reliable data for all Korean citizens. We found that handgrip strength and anemia had a clear correlation. The proportion of those with anemia and weaker handgrip strength was higher than that found for the other groups. Even after controlling for sociodemographic factors, there was a clear correlation between handgrip strength and anemia. Furthermore, after controlling for individual lifestyle habits, such as smoking, alcohol consumption, nutritional and disease-related factors, handgrip strength and anemia showed a very significant association. In comparing the group with and without anemia, indices such as BMI, level of daily workout, and iron intake per day showed significant differences. One noticeable finding was that the prevalence rate of anemia was higher for non-smokers and those with a lower consumption of alcohol (Table 1). This may be due to the fact that females formed a greater proportion of those with anemia.

The relationship between anemia and handgrip strength can be explained by a biological mechanism as reported in previous studies. The low strength can be explained by weakness and fatigue which can increase the risk of disability in these individuals [53]. Hemoglobin, in the red blood cells, is responsible for transporting oxygen to various parts of the body. Therefore, the lower blood concentration of Hb means lower oxygen is transported, and this can increase the risk of various mobility disorders along with fatigue [54]. Because the hemoglobin concentration plays an important role in the oxygenation of the blood [20, 21], anemia, defined by levels of Hb, represents a reduction in oxygenation function. Hypoxia due to anemia can reduce the oxygen supply to the muscles and thereby affect the strength of the muscles [4].

We performed subgroup analyses by sex and age; these were predicted to affect anemia. In the subgroup by sex, the correlation between handgrip strength and anemia was higher for males compared to females. For males, indices such as BMI, level of daily workout, and comorbid diseases had significant relationships with anemia (Table 2). Additionally, in the subgroups by age, the association between handgrip strength and anemia was more pronounced in participants ≥65 years compared to those <65 years Although the proportion of females with anemia was far higher in the group <65 years, the male/female distinction did not show large differences in those ≥65 years. It was also found that BMI, level of daily workout, and iron intake per day were significantly related to anemia in both groups (Table 3).

Previous studies have shown significant relationships between anemia and handgrip strength in only females, rather than males. These included a study that found significant differences in Hb levels in males among the South African population >40 years old [55] and a study that found an association between anemia and handgrip strength in the Brazilian female population >60 years old [12]. However, this study showed a higher OR of anemia in the weak handgrip strength group compared to the strong handgrip strength group in both males and females, and this association was greater in males compared to females. The differences may be due to heterogeneity of the aging population with regards to race, living environments, and health problems, all factors that can affect Hb levels [56].

Furthermore, previous studies on the elderly have found a clear association between handgrip strength and anemia. According to the research by Hirani V et al., the decline in Hb concentration in Australian elderly participants was directly related to the decline in handgrip strength [57]). Pennix et al also reported that patients with anemia (defined by the Hb level among elderly participants >65 years), had far lower handgrip strength compared to patients without anemia [4]. Moreover, research on the association between anemia and handgrip strength in the elderly population >90 years old [55, 58] and research showing an association between anemia, handgrip strength, and lower-body strength in the elderly >100 years old [59] demonstrated that the association may increase as age increases. The reason could be that the reduction in handgrip strength due to aging can represent the overall health of the elderly [60]. In particular, because the elderly often have higher blood concentration of C-reactive protein, acute or chronic inflammation can result in an even larger physical decline [4, 5]. Therefore, the maintenance and management of handgrip strength are important especially in the elderly population, and it will be important to identify factors that affect handgrip strength [61].

There are some limitations to keep in mind while interpreting these results. **First**, because this study used a cross-sectional study design based on the KNHANES, caution must be taken in interpreting a causal relationship between the variables. In other words, we cannot assume that handgrip strength is an independent causative factor of anemia or an epiphenomenon. **Second**, many variables measured at a single time point were used to assess the effects of handgrip and anemia. This may negatively affect the accuracy of the data to some extent. The socio-demographic characteristics of the study population were collected through surveys. Therefore, this may have caused a recall bias. **Third**, there is a limitation in identifying the risk factors for anemia because of insufficient nutritional data. Micronutrient deficiencies including folate and vitamin B12 are main causes of anemia [62]; however, the study lacks data on nutritional status. Therefore, it was difficult to clearly consider the effect of diseases that could be related to anemia. Fourth, because KNHANES only considered Hb levels to define anemia, it was difficult to consider the types or causes of anemia. Therefore, additional research is necessary on variables that can affect anemia; it is anticipated that the results of such a study would have clinical applicability. Finally, we did not process missing data using methods such as multiple imputations (MI) and could not adjust for more stringent semantic thresholds, which could only be solved by well-designed randomized controlled trials. However, I believe that the results obtained with missing data excluded were no different from the results obtained if the missing data had been processed using MI. This is because of the nationally representative sample included in this study. The strict important thresholds can be addressed later using well-designed clinical studies.

Despite these limitations, this research has the following advantages. **First**, this study is the first to examine the correlation between handgrip strength and anemia in Korean adults. Previous studies on the relationship between handgrip strength and anemia have been controversial [4, 12, 22, 23]. Previous studies have shown significant relationships between anemia and handgrip strength in only females [12, 55] or the elderly [55, 57–59]. In our study, we found that regardless of sex and age, there was a significant association between handgrip strength and anemia. **Second**, the study was based on reliable data obtained from a nationwide survey targeting a sample representative of the general Korean population [24]. Therefore, the results of this study showed that the relationship between handgrip strength and anemia was very reliable. **Third**, various covariates such as sociodemographic factors, lifestyle factors and illness related factors that could affect the association between anemia and handgrip strength adjusted in model. **Finally**, this study divided subgroups according to gender and age, and compared the characteristics of the anemia population. Through this, it was possible to determine the high prevalence of anemia in each subgroup.

Through this study, we found that regardless of sex and age, there was a significant association between handgrip strength and anemia. Therefore, handgrip strength can be used as a risk factor when screening for anemia in patients, and can be used in clinical practice for testing for anemia in the elderly, in epidemiological studies, or during treatments[12]. In particular, handgrip strength may be a useful screening tool for the elderly who are more likely to develop other diseases associated with anemia and for males who have lower prevalence of anemia than females. Further study is needed to identify and clinically track populations more likely to develop anemia by measuring handgrip strength.

## Conclusion

In conclusion, there was a strong association between handgrip strength and anemia. In particular, the association between handgrip strength and anemia was more likely to occur in males and those who were older than 65 years.
